# Cathepsin D promotes polarization of tumor-associated macrophages and metastasis through TGFBI-CCL20 signaling

**DOI:** 10.1038/s12276-024-01163-9

**Published:** 2024-02-01

**Authors:** Seul Gi Lee, Seon Min Woo, Seung Un Seo, Chan-Hyeong Lee, Moon-Chang Baek, Se Hwan Jang, Zee Yong Park, Simmyung Yook, Ju-Ock Nam, Taeg Kyu Kwon

**Affiliations:** 1 Department of Immunology, School of Medicine, Keimyung University, Daegu, Republic of Korea; 2 Department of Microbiology, Immunology, and Cancer Biology, University of Virginia, Charlottesville, VA USA; 3 Department of Molecular Medicine, CMRI, Exosome Convergence Research Center (ECRC), School of Medicine, Kyungpook National University, Daegu, Republic of Korea; 4 School of Life Sciences, Gwangju Institute of Science and Technology, Gwangju, Korea; 5 Department of Biopharmaceutical Convergence, Sungkyunkwan University, Suwon, Republic of Korea; 6 Department of Food Science and Biotechnology, Kyungpook National University, Daegu, Republic of Korea; 7 Center for Forensic Pharmaceutical Science, Keimyung University, Daegu, Republic of Korea

**Keywords:** Cancer microenvironment, Epithelial-mesenchymal transition, Targeted therapies

## Abstract

M2-like tumor-associated macrophages (TAMs) are risk factors for cancer progression and metastasis. However, the mechanisms underlying their polarization are still not fully understood. Although cathepsin D (Cat D) has been reported as a procarcinogenic factor, little is known about the functional role of Cat D in the tumor microenvironment (TME). This study aimed to explore the effect and molecular mechanisms of Cat D in the TME. Cat D knockout (KO) altered the cytokine secretion pattern and induced TAM reprogramming from the M2 to M1 subtype, thereby preventing epithelial-mesenchymal transition and tumor metastasis. Mechanistically, we identified transforming growth factor beta-induced protein (TGFBI) as a Cat D target protein that is specifically associated with TAM polarization. Elevated TGFBI expression in Cat D KO cancer cells resulted in a decline in M2-like TAM polarization. Our RNA-sequencing results indicated that the cancer cell-secreted chemokine CCL20 is a major secretory chemokine for Cat D-TGFBI-mediated TAM polarization. In contrast, Cat D overexpression accelerated TAM polarization into M2-like cells by suppressing TGFBI expression. In addition, the double Cat D and TGFBI KO rescued the inhibitory effects of Cat D KO on tumor metastasis by controlling TAM and T-cell activation. These findings indicated that Cat D contributes to cancer metastasis through TGFBI-mediated TAM reprogramming. Cat D deletion inhibits M2-like TAM polarization through TGFBI-mediated CCL20 expression, reprogramming the immunosuppressive TME. Our results open a potential new avenue for therapy focused on eliminating tumor metastasis.

## Cathepsin D: A Game Changer in Cancer Metastasis and TAM Polarization

Understanding how certain immune cells in tumors behave can give us insights into cancer growth. TAMs can either support the tumor or help the body fight it, depending on signals they receive from their surroundings. This study focuses on an enzyme called Cathepsin D, which is involved in several cancers and influences how TAMs act within tumors. Researchers conducted an experiment using mice and various cancer cell types to see how Cat D influences TAM behavior and cancer growth. They genetically altered cancer cells to lack Cat D and observed the effects on tumor growth and the immune response in mice. The results showed that removing Cat D from cancer cells led to a decrease in tumor growth and changes in TAMs made them more effective at fighting the tumor.

This summary was initially drafted using artificial intelligence, then revised and fact-checked by the author.

## Introduction

Tumor-associated macrophages (TAMs) are the most abundant immune cell type in tumors and exhibit high phenotypic heterogeneity in response to environmental signals. In many tumor types, TAMs exhibit similarities to M2-like macrophages and suppress T-cell activation, contributing to enhanced tumor progression to malignancy^1,2^. Alternately, TAMs also show an M1-like phenotype that is characterized by the secretion of the inflammatory cytokines IL-1β and TNF-α. Previous reports have indicated that the M2-to-M1 phenotypic macrophage transition is linked to the suppression of metastatic tumors and epithelial–mesenchymal transition (EMT)^3,4^, with a complex interaction involved. Once cancer-mediated pathogens and cytokines promote the recruitment of monocytes into the tumor, these infiltrated TAMs can, in turn, secrete various pro- and anti-tumorigenic cytokines that regulate extracellular matrix protein (ECM) remodeling, angiogenesis, metastasis, and immune evasion^5,6^. Thus, controlling TAM transition is a potential combination therapy for treating the progression and metastasis of various types of cancer.

Cathepsin D (Cat D) has been implicated in the pathogenesis of breast, ovarian, and gastric cancers^7–9^. Elevated Cat D levels have been found in patients with breast cancer, and downregulation of this molecule ameliorated cancer proliferation, angiogenesis, and metastasis^10–12^. Our earlier study revealed that inhibiting Cat D in cancer cells appeared to sensitize them to anticancer drugs by regulating Bcl-xL stabilization^13^. In addition, Cat D is active in extralysosomal locations, including the cytosol and extracellular environment^14^. Furthermore, previous studies have shown that mature Cat D (m-Cat D) cleaves and activates BH3 interacting domain death agonist (BID), resulting in apoptosis, and acts as an extracellular ligand to promote cell proliferation^15^. However, to date, the function of Cat D in the tumor microenvironment (TME) and its precise mechanisms remain largely unknown. Herein, we studied the contribution of Cat D to EMT and TAM polarization and their antitumor activity and highlighted the molecular mechanisms underlying this effect.

## Materials and methods

### Animals

SCID female mice were injected with 2 × 10^6^ MDA-MB-231-parental or genetically modified MDA-MB-231 cells expressing Cat D KO (CKO), TGFBI KO (TKO), and double KO (DKO) vectors into the tail vein. Bioluminescence images were obtained using an IVIS system after injection of 2 × 10^6^ luciferase-expressing MDA-MB-231 (Luc-MDA-MB-231) cells into female SCID mice. In some experiments, we injected 2.5 mg/kg cilengitide for 2.5 weeks after cancer cell injection. C57BL/6 female mice were inoculated with 1 × 10^6^ E0771 parental or genetically modified E0771 cells into the mammary fat pad or the tail vein. Body weight was recorded every 3 days for the entire experimental period. On the final experimental day, subcutaneous tumors were collected to analyze immune populations in these tumors. All animal experiments were approved by the Institutional Animal Care Committee of Keimyung University (approval number: KM-2021-09R1). The study was performed in compliance with the ARRIVE guidelines, and all methods were implemented according to relevant guidelines and regulations.

### Cell culture

MDA-MB-231, Caki cancer cell lines, and THP-1 monocytes were obtained from the American Type Culture Collection (ATCC). E0771, 4T1, B16F10, and CT26 cancer cell lines were purchased from Korea Cell Line Bank (Seoul, Korea). All cancer cells were maintained in DMEM with 10% fetal bovine serum (FBS) and antibiotics (1% penicillin/streptomycin) at 37 °C in a humidified 5% CO_2_ incubator.

### Polarization of THP-1 cells

For preparation of conditioned media (CM), Caki and MDA-MB-231 cancer cells were incubated in DMEM (with 10% FBS) for 48 h, CM was collected, and then, cell debris was cleared by centrifugation at 500 × *g* for 10 min. For some functional experiments, cancer cells were treated with either 20 µg/ml rcTGFBI or 0.5 µM cilengitide for 48 h. The resulting CM was stored at −70 °C for subsequent experiments. For induction of THP-1 polarization into TAMs, THP-1 monocytes were treated with 50 nM phorbol 12-myristate 13-acetate (PMA) and then cultured with CM for 48 h. PMA-induced macrophages were treated with 10 ng/ml LPS and 20 ng/ml IFN-γ or 20 ng/ml IL-4 and 20 ng/ml IL-13, which were used as M1 and M2 macrophages, respectively.

### siRNA interference

For transient silencing of Cat D and CCL20 in Caki and MDA-MB-231 breast cancer, Cat D siRNA (sc-29239) and CCL20 (sc-60000) siRNA were obtained from Santa Cruz Biotechnology (Dallas, TX, USA). The control siRNA was purchased from Bioneer (Daejeon, Korea). All siRNAs were transfected using Lipofectamine® RNA iMAX Reagent (Invitrogen, Carlsbad, CA, USA). After transfection at 6 h, the medium was changed to complete growth medium.

### Plasmid construction and generation of KO stable cells

A single guide (sgRNA) was designed to knock out Cat D and TGFBI using the CRISPR‒Cas9 tool. In some experiments, MDA-MB-231 and E0771 breast cancer cells were transfected with Cat D KO and TGFBI KO vectors. The following sequences were used: human Cat D 5′-CAC CGA TGG GCC CCT CGG TCA CGG C-3′, human TGFBI 5′-CAC CGG TGC TGA AGC CAT CGT TGC G-3′, mouse Cat D 5′-CAC CGC GTC CTC CTT CGC GAT TAT C-3′, and mouse TGFBI 5′-CAC CGG CAC GCC GTT GGT CGC A-3′. The KO vector was constructed into pSpCas9 (BB)-2A-Puro (PX459) (Addgene, Watertown, MA, USA) and transfected into cancer cells using Lipofactor-pMAX (Aptabio, Yongin, Korea). After 2 days, resistant cells were selected with 0.5 μg/mL puromycin, and single-cell clones were picked and analyzed by western blotting.

### Cancer migration, colony formation, and invasion assays

For the migration assay, cancer cells were cultured until reaching 100% confluence, after which a scratch was made through the confluent cell monolayer using a 200 µl pipette tip. The cells were photographed under a microscope at the indicated time points. The relative migration rate was calculated using the following formula: covered area (fold) = average migration area—average no migration area. In the colony formation assay, cells were seeded at a density of 50–100 cells/well and cultured for 5–7 days. On the final day of the experiment, the cell colonies were fixed in 4% paraformaldehyde (PFA) and stained with 0.25% crystal violet. The invasion assay was performed in the chambers of 8-μm Transwell inserts. The cells were seeded into the top chamber and left to migrate for 24 h. Afterward, the inserts were cleaned with cotton swabs, and migrated cells were fixed and stained.

### Flow cytometry

The polarized THP-1 macrophages were harvested, washed, and then labeled with APC-CD204 (Cat. FAB2708B; R&D), PE-CD86 (Cat. 553692, R&D), or PE-CD163 (Cat. FAB1607A; R&D) and analyzed using a BD Accuri™ C6 flow cytometer (BD Biosciences, San Jose, CA, USA). Infiltrated macrophages were isolated from CON, CKO, and DKO tumors. The tumors were dissociated with collagenase type I, and CD45+ hematopoietic cells were sorted using a CD45 microbead. Magnetic-activated cell sorting was performed according to the manufacturer’s instructions (Miltenyi Biotec, Bergisch Gladbach, Germany). CD45+ microbead-labeled cells were stained with the following antibodies: Cy7-CD45, FITC-CD11b, eFluor450-F4/80, PE-CD86, APC-CD206, Cy7-CD45, FITC-CD3, Cyanine-CD4, and eFluor450-CD8. After staining, the cells were washed with phosphate-buffered saline (PBS) and then analyzed using the CytoFLEX system (Beckman Coulter, Brea, CA, USA).

### Cytokine array

The culture medium was collected from parental Caki, Cat D KO Caki, and polarized THP-1 cells. The debris was removed by centrifugation for further analysis using the human XL cytokine array kit (Cat. ARY022; R&D Systems) according to the manufacturer’s protocol. The spot intensities were evaluated using ImageJ software.

### Western blot analysis

Lysate preparation and western blot analysis were performed as previously described^16^. Total proteins were separated using 8–12% sodium dodecyl sulfate‒polyacrylamide agarose gel electrophoresis (SDS‒PAGE) and transferred to nitrocellulose membranes. The primary antibodies were as follows: Bcl-xL (Cat. sc-634; Santa Cruz Biotechnology, Dallas, TX, USA), Cathepsin D (Cat. sc-377299; Santa Cruz Biotechnology), Arg1 (Cat. sc-18354; Santa Cruz Biotechnology), CCL20 (Cat. sc-51744; Santa Cruz Biotechnology), E-cadherin (Cat. ab40772; Abcam, Burlingame, CA, USA), TGFBI (Cat. ab189778; Abcam), Cox2 (Cat. ab15191; Abcam), vimentin (Cat. 3390; Cell Signaling Technology, Danvers, MA, USA), Snail (Cat. 3879; Cell Signaling Technology), Src (Cat. 8056; Cell Signaling Technology), p-Src (Cat. 2101; Cell Signaling Technology), STAT1 (Cat. 9172; Cell Signaling Technology), p-STAT1 (Cat. 9171; Cell Signaling Technology), Myc-Tag (Cat. 2276; Cell Signaling Technology) and actin (Cat. A5441, Sigma‒Aldrich, St Louis, MO, USA). Subsequently, the membranes were incubated with HRP-conjugated anti-mouse or anti-rabbit secondary antibodies for 1 h at room temperature. The signals were detected and quantified using an iBright CL750 imaging system (Invitrogen, Carlsbad, CA, USA).

### Quantitative real-time PCR (qRT‒PCR)

Total RNA was extracted using TRIzol reagent (Life Technologies, Gaithersburg, MD, USA), and complementary DNA (cDNA) was synthesized. Afterward, real-time PCR was performed with SYBR Fast qPCR Mix (TaKaRa Bio, Inc., Shiga, Japan) using Thermal Cycler Dice® Real-Time System III (TaKaRa Bio, Inc., Shiga, Japan). The relative gene expression levels were determined by the 2^−ΔΔCt^ method, and β-actin was used as the control gene. The primer sequences are listed in Supplementary Table 1, and β-actin was used as the control gene.

### Immunofluorescence staining

Differentiated THP-1 macrophages were fixed in 4% PFA, permeabilized with 0.25% Triton X-100, and blocked with blocking buffer containing 1% BSA in PBS. The cells were incubated with a CD163-specific primary antibody (Cat. sc-20066; Santa) O/N at 4 °C and stained with a secondary antibody (Alexa Fluor^®^ 488) under the same conditions. The samples were then mounted using DAPI-containing mounting solution (Vector Laboratories, Burlingame, CA, USA) and imaged using a confocal laser microscope (Carl Zeiss, Jena, Germany).

### Immunohistochemistry

Metastatic liver and lung tissues were fixed with 4% PFA, processed, and embedded in paraffin as described in a previous report^16^. Tissue Section (7 μm thick) were stained with hematoxylin and eosin (H&E) and photographed randomly under a microscope.

### Capillary reverse-phase liquid chromatography (LC)-tandem mass spectrometry (MS/MS) and data analysis

Total proteins were isolated from parent and Cat D KO Caki cancer cells according to a previously described method^17,18^. Briefly, cells were loaded onto silica capillary columns containing 7.5 cm of 5 lm particle Aqua C18 reversed-phase column material. The column was placed in-line with an Agilent HP1100 quaternary LC pump, and a splitter system was used to achieve a flow rate of 250 nL/min. Peptides were eluted and electrosprayed into an LTQ Ion Trap mass spectrometer (Thermo Finnigan, Palo Alto, CA, USA). MS/MS spectra were individually matched with the human IPI protein using the TurboSEQUEST and SEQUEST Cluster Systems.

### Human tumor biopsies

All normal and renal cancer tissues were obtained from Keimyung University Dongsan Hospital. This study followed the medical research protocols of and was approved (approval number: IRB-2019-11-040) by the Research Ethics Committee of Keimyung University. Cat D mRNA expression in breast and renal tumors of adjacent tissues was analyzed from the TCGA database by the GEPIA website^19^.

### Statistical analysis

Statistical analyses for all experiments were performed using SPSS ver. 20.0 (SPSS, Inc., Chicago, IL, USA). Statistical differences between groups were analyzed using a two-sided t-test, and statistical significance was set at *p* < 0.05.

## Results

### Loss of Cat D induces suppressed malignant behavior of cancer cells

**Fig. 1:**
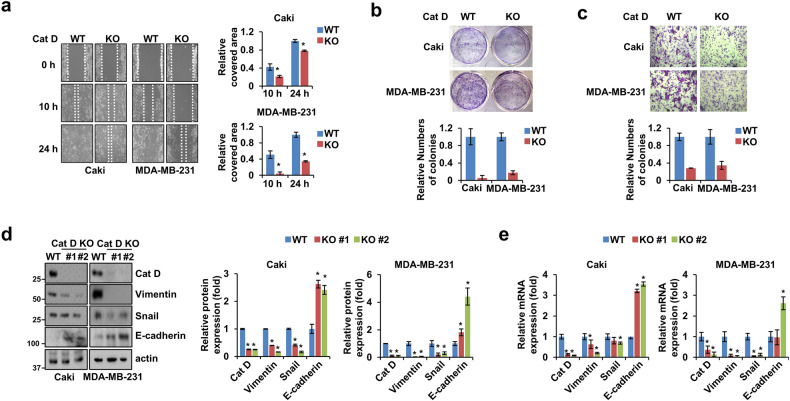
Suppressed metastatic potential in Cat D KO cancer cells. **a**–**e** Parent cancer and Cat D KO stable cancer cells are referred to as “WT” and “KO,”, respectively, unless otherwise stated. Cells were wounded and imaged under a microscope at the indicated time points, and the Caki and MDA-MB-231 cancer cell migration areas were quantified (**a**). Original magnification = 10×. Colonies (**b**) and invaded (**c**) Caki and MDA-MB-231 cells were photographed and counted (bottom). The protein (**d**) and mRNA (**e**) expression of Cat D, vimentin, snail, and E-cadherin in the WT and KO cells. #1 and #2 indicate the individual colonies selected and picked after vector transfection. Actin was used as the control gene. Error bars represent the ± SEM. *p < 0.01 in a two-sided t-test.

First, we examined whether the loss of Cat D affected the metastatic properties of Caki and MDA-MB-231 cancer cells. The Cat D knockout (KO) cancer cells showed reduced migration, colony formation, and invasion compared with the parent cancer cells (Fig. 1a–c). CRISPR/Cas9-mediated KO of Cat D in Caki cancer cells was verified by DNA sequencing and protein expression analysis (Supplementary Fig. 1a and Fig. 1d). Consistent with the suppressed motility of cancer cells, the mRNA and protein expression of vimentin and snail were markedly worsened in the KO cells compared with the control cells, while the opposite result was observed for E-cadherin expression (Fig. 1d, e). In addition, we confirmed the effects of transient Cat D knockdown in both Caki and MDA-MB-231 cancer cells. The Cat D siRNA-transfected cells (Cat D siRNA) showed a decline in migration and colony formation compared with the control siRNA-transfected cells (Supplementary Fig. 1b, c). Moreover, the protein and mRNA expression levels of vimentin and snail were reduced, whereas the opposite result was confirmed for E-cadherin expression in the Cat D siRNA-transfected cancer cells (Supplementary Fig. 1d, e). These results indicate that Cat D plays a role in the metastatic properties of cancer cells.

### Loss and gain of Cat D expression regulate transforming growth factor beta-induced protein (TGFBI) accumulation

**Fig. 2:**
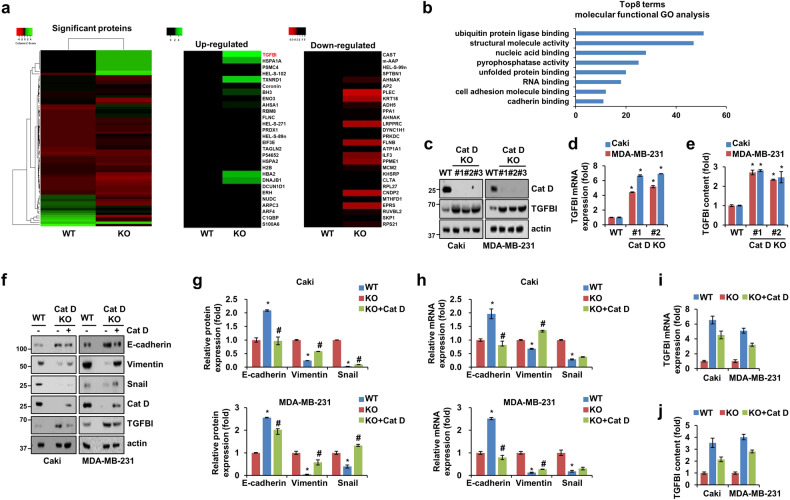
Cat D negatively regulates the expression and secretion of TGFBI. **a, b** Heatmap of significant proteins (**a**) and molecular functional GO analysis (**b**) based on LC‒MS/MS proteomics. **c**, **d** The protein and mRNA expression of the indicated genes in Caki and MDA-MB-231 cells. **e** Secretory TGFBI content in CM collected from the WT and KO mice was determined using an enzyme-linked immunosorbent assay (ELISA). **f**–**j** KO cells were transfected with a Cat D overexpression vector (OE). WT and KO cells were transfected with the same amount of an empty vector. Representative western blot images of the indicated proteins in the cells transfected with Cat D OE or empty vector (**f**). Protein (**g**) and mRNA (**h**) expression of EMT markers in the transfected cells. TGFB mRNA (**i)** and secretory (**j**) levels in Caki and MDA-MB-231 cancer cells. Error bars represent the ± SEM. *p < 0.01 compared with the WT cells. ^#^*p* < 0.01 com*p*ared with the KO cells.

To determine the possible reason for the changes in metastatic properties in Cat D KO cancer cells, we analyzed total protein using proteomic technology. Hierarchical clustering analysis identified distinct protein expression patterns. Among these proteins, 136 significant proteins (at least >1.5-fold change and *p* < 0.05) were found, and we identified 70 upregulated and 66 downregulated proteins in the Cat D KO cancer cells compared with those in the control cells (Fig. 2a). Among the significant proteins, we found that the transforming growth factor beta-induced (TGFBI) protein showed the highest increase (7.6-fold) in the Cat D KO cells compared with the control cells. TGFBI, an ECM protein, has been reported to interact with various receptors and cancers^20,21^. Proteins related to structural molecule activity and cell adhesion molecule binding were greatly enriched and altered in the Cat D KO cells (Fig. 2b). Elevated TGFBI expression following Cat D deletion was confirmed in both Caki and MDA-MB-231 cancer cells (Fig. 2c). Furthermore, the mRNA expression and secretion of TGFBI were increased in the Cat D deletion cancer cells (Fig. 2d, e). Accordingly, Cat D knockdown also induced the upregulation of protein and mRNA expression and the secretion of TGFBI in both Caki and MBA-MB-231 cancer cells (Supplementary Fig. 2a–c). Alpha-V beta-3 (αvβ3), one of the receptors for TGFBI, displayed either no significant difference or lower expression in Caki and MDA-MB-231 Cat D KO cells compared to their control cells (Supplementary Fig. 2d, e). These results suggest that extrinsic receptor signaling may not play a pivotal role in the Cat D deletion-induced regulation of TGFBI expression. We examined whether the gain-of-function of Cat D could regulate the expression of TGFBI and EMT markers in cancer cells. The expression of E-cadherin decreased, while that of vimentin and snail was rescued in these add-back cells compared with those in the Cat D KO cells (Fig. 2f–h). Additionally, the addition of Cat D restored the protein, mRNA, and secretory levels of TGFBI in both Caki and MDA-MB-231 Cat D KO cancer cells (Fig. 2f, i, j). These results indicate that the loss and gain of Cat D strongly regulate TGFBI and EMT marker expression.

### Loss of Cat D limits cancer metastasis, and Cat D-mediated TGFBI has no strong effects in immune-deficient mice

**Fig. 3:**
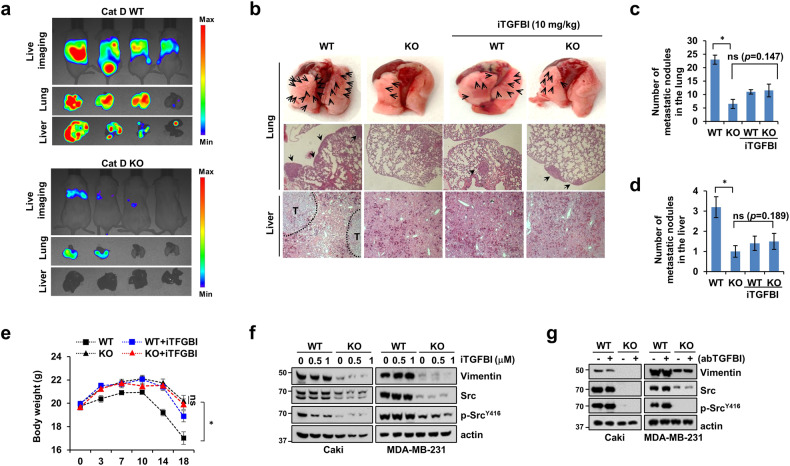
Cat D KO cancer-bearing mice exhibit inhibition of metastasis to the lungs and liver. **a** Luc-MDA-MB-231 breast cancer cells were tail-vein injected into SCID mice. **b**–**e** MDA-MB-231 breast cancer cells were inoculated and subsequently treated with the TGFBI inhibitor cilengitide (referred to in the text as “iTGFBI”) for 2.5 weeks through tail vein injection. Representative lung and liver H&E images obtained from the WT and KO cancer-bearing mice receiving either PBS or iTGFBI. Arrows and “T” indicate tumor cells. Original magnification = 10× (**b**). Visual metastatic nodules in the lung (**c**) and liver (**d**) and mouse body weight. **f**, **g** Caki and MDA-MB-231 cells were treated with iTGFBI (**f**) or abTGFBI (**g**). Error bars represent the ± SEM. *p < 0.01 in a two-sided *t* test.

Next, we investigated whether blocking TGFBI in Cat D KO tumors controls tumor metastasis in immunodeficient (SCID) mice. To this end, we employed cilengitide (hereafter referred to as iTGFBI), a selective antagonist of αvβ3 and vβ5 integrins that binds to TGFBI. The Cat D KO cancer-bearing mice showed decreased metastasis to the liver and lungs and loss of body weight compared with the control cancer-bearing mice (Fig. 3a–e). Moreover, iTGFBI reduced metastasis and body weight loss in the control cancer-bearing mice. However, body weight and lung and liver metastases did not differ considerably between the iTGFBI-treated and untreated Cat D KO cancer-bearing mice (Fig. 3b–e). Furthermore, treatment with iTGFBI or a neutralizing antibody did not affect the expression of vimentin and Src phosphorylation in either the control or Cat D KO cancer cells (Fig. 3f, g). These results suggest that the functional inhibition of TGFBI did not completely rescue the inability of Cat D KO to metastasize in cancer cells.

**Fig. 4:**
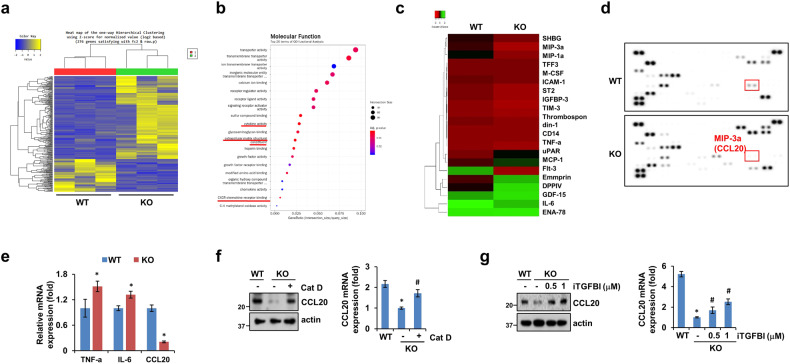
Cat D KO cancer cells secrete distinct cytokine profiles compared with those in control cancer cells. **a**, **b** Expression heatmap (**a**) and molecular function (**b**) in WT and Cat D KO Caki cancer cells based on RNA-sequencing data. **c**–**g** Expression heatmap (**c**) and western blot (**d**) images showing changes in cytokine expression in CM from WT and Cat D KO Caki cells. mRNA expression in WT and Cat D KO cells (**e**). CCL20 protein and mRNA in Caki cells (**f**, **g**). Error bars represent the ± SEM. *p < 0.01 compared with the WT cells. ^#^*p* < 0.01 com*p*ared with the Cat D KO cells.

Given the suppressed metastasis and minor effects of TGFBI in the Cat D KO cancer-bearing mice, we hypothesized that Cat D-mediated TGFBI upregulation might have an additional and closely related role in the TME. To determine the effect of TGFBI, we performed RNA sequencing on control and Cat D KO Caki cells (Fig. 4a). Among the molecular functions modulated in the Cat D KO cancer cells, we found differences in cytokine activity, extracellular matrix structural constituents, and CXCR receptor binding (Fig. 4b). We further confirmed a distinct cytokine secretion pattern between the control and Cat D KO cancer cells (Fig. 4c, d and Supplementary Table 2). Notably, CCL20, a chemokine that induces macrophage polarization into the M2 subtype, was significantly reduced in the Cat D KO cancer cells at both the protein and mRNA levels (Fig. 4d, e). In addition, higher TNF-α and IL-6 expression was observed in the Cat D KO cancer cells (Fig. 4e). CCL20 expression was rescued by Cat D addition and increased in a dose-dependent manner in response to iTGFBI treatment of Cat D KO cancer cells (Fig. 4f, g). These results imply that Cat D-mediated TGFBI may be linked to TAM polarization, possibly through cytokine secretion.

### Cat D KO cancer cells suppress M2-like polarization of TAMs by regulating TGFBI expression

Intrigued by the distinct cytokine patterns, we next investigated whether Cat D KO cancer cells could facilitate reprogramming of the TAM subtype with antitumor functions. We first assessed whether IL-4- and IL-13-induced M2 macrophages exhibite relatively lower levels of p-STAT1 and TNF-α and higher levels of Cox2 and Arg1 than LPS- and IFNγ-induced M1 macrophages (Supplementary Fig. 3a). To investigate the role of Cat D in TAM polarization, we established an appropriate dose of conditioned medium (CM) collected from the control or Cat D KO cancer cells in which the ratio of CM to control medium was 2:1 for inducing M2-like TAMs (Supplementary Fig. 3b, c). The THP-1 macrophages cultured with CM at a ratio of 2:1 exhibited elevated CD163 and Arg1 expression and reduced CD64, TNF-α, and IL-1β expression compared with the THP-1 cells cultured with CM at a ratio of 1:1.

**Fig. 5:**
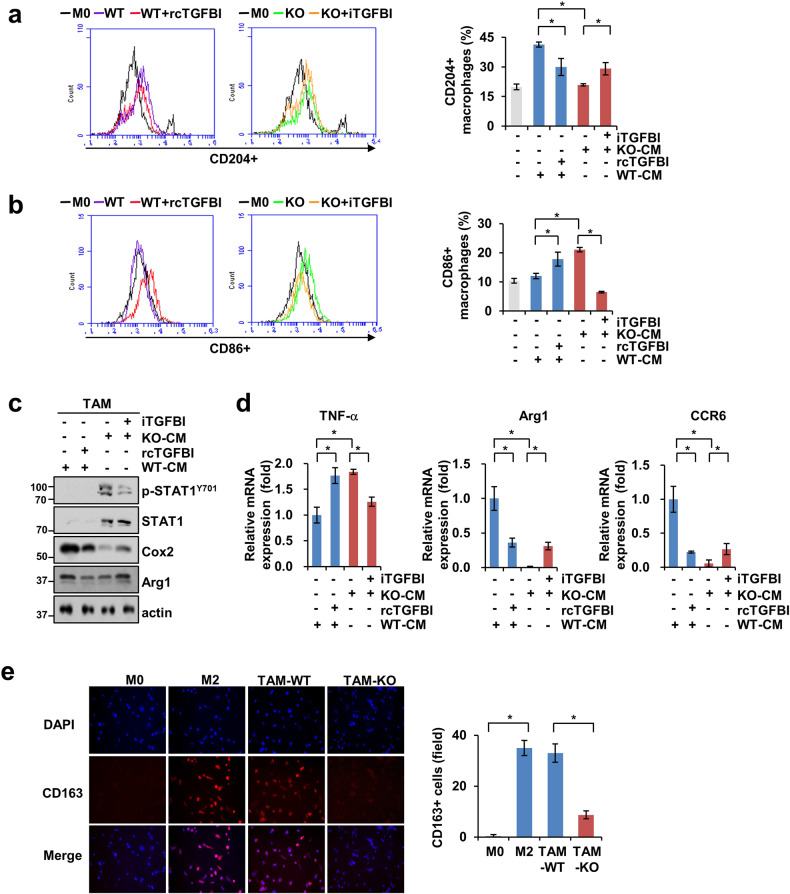
Loss of Cat D induces the reprogramming of THP-1 polarization into an M1-like subtype. **a**–**d** THP-1 cells were cultured with either WT-CM or KO-CM from Caki cancer cells receiving rcTGFBI or iTGFBI for 2 days. CD204^+^ (**a**) and CD86^+^ (**b**) THP-1 cells were determined by FACS analysis. Protein (**c**) and mRNA (**d**) expression of the indicated genes from polarized THP-1 cells. **e** THP-1 macrophages were polarized with WT-CM or KO-CM from Caki cancer cells for 2 days, and then, CD163^+^ THP-1 (red) was observed by Immunofluorescence staining. The stained cells were counted under a fluorescence microscope at 20× magnification. IL-4- and IL-13-induced M2 macrophages were used as positive controls. Error bars represent the ± SEM. *p < 0.01 in a two-sided *t* test.

Interestingly, CM from the Cat D KO (KO-CM) Caki cancer cells suppressed the polarization of THP-1 macrophages into CD204^+^ TAMs and promoted CD86^+^ TAMs compared with CM from the control cells (WT-CM) (Fig. 5a, b). Furthermore, treatment with rcTGFBI reduced CD204^+^ TAMs induced by WT-CM, and in contrast, iTGFBI treatment increased CD204^+^ TAMs induced by KO-CM (Fig. 5a). The opposite results were observed for CD86^+^ TAMs, which were increased and decreased by rcTGFBI and iTGFBI in the THP-1 cells cultured with WT-CM and KO-CM, respectively (Fig. 5b). pY-STAT1 expression was increased and Cox2 and Arg1 were decreased in the TAMs cultured with KO-CM compared with those cultured with WT-CM (Fig. 5c). In addition, rcTGFBI decreased the expression of Cox2 and Arg1 in the TAMs cultured with WT-CM, whereas iTGFBI increased Cox2 and Arg1 and reduced the expression of pY-STAT1 in the TAMs cultured with KO-CM (Fig. 5c). The mRNA expression of TNF-α was increased in the THP-1 cells cultured with KO-CM compared with that in the THP-1 cells cultured with WT-CM. In addition, TNF-α mRNA expression was upregulated and downregulated in the THP-1 cells cultured with WT-CM and KO-CM and treated with rcTGFBI and iTGFBI, respectively (Fig. 5d). In contrast, the expression of Arg1 and CCR6 was diminished in the THP-1 cells cultured with KO-CM and was downregulated and upregulated by rcTGFBI and iTGFBI treatment, respectively (Fig. 5d). Furthermore, we found that the staining intensity of CD163, a marker of M2-like TAMs, was lower in the THP-1 cells cultured with KO-CM than in those cultured with WT-CM (Fig. 5e). In addition, an increase in M2-like TAM polarization was observed in the THP-1 cells cultured with Cat D add-back Caki and MDA-MB-231 cells compared with that in the Cat D KO cells (Supplementary Fig. 4). Our observations indicated that Cat D deletion in cancer cells skewed TMA polarization to the anticancer M1 phenotype through TGFBI overexpression.

### Cat D mutations result in suppressed M2-like TAM polarization

**Fig. 6:**
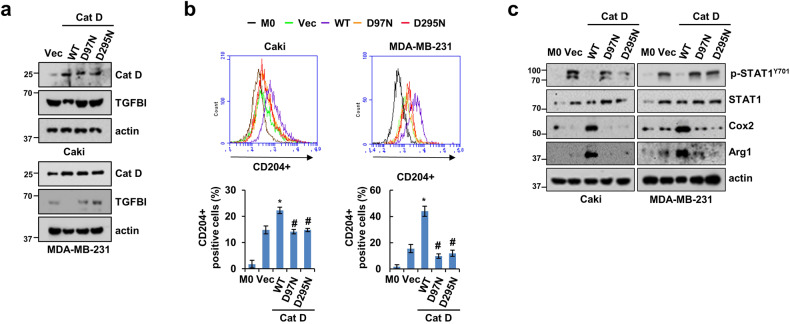
Cat D mutations predominantly decrease the polarization of THP-1 cells into M2-like TAMs. **a** Protein expression of Cat D and TGFBI in Caki and MDA-MB-231 cells transfected with the indicated Cat D WT and mutants (D97N and D295N). **b**, **c** THP-1 cells were polarized with CM from the Caki and MDA-MB-231 cells expressing Cat D WT and mutants. CD204^+^ THP-1 cells were determined by FACS analysis (**b**). Representative western blot images of THP-1 cells primed with the Caki and MDA-MB-231 cells expressing Cat D WT and mutants (**c**). Error bars represent the ± SEM. *p < 0.01 compared with the control cells. ^#^*p* < 0.01 com*p*ared with the Cat D WT-transfected cells.

We generated catalytically inactive Cat D by point mutation of the aspartate residues D97 and D295. We first assessed whether the two Cat D mutations upregulated TGFBI expression in both Caki and MDA-MB-231 cells compared with that in the Cat D WT-transfected cells (Fig. 6a). CD204^+^ TAM polarization and Cox2 and Arg1 expression levels in the THP-1 cells cultured with Cat D mutation cancer cells were dramatically decreased, whereas those of pY-STAT1 were increased relative to those of the THP-1 cells cultured with Cat D WT cancer cells (Fig. 6b, c). These findings suggest that the catalytic activity of Cat D is linked to the regulation of TGFBI expression and the polarization of TAMs.

### CCL20 is a key chemokine in Cat D-mediated TAM polarization

**Fig. 7:**
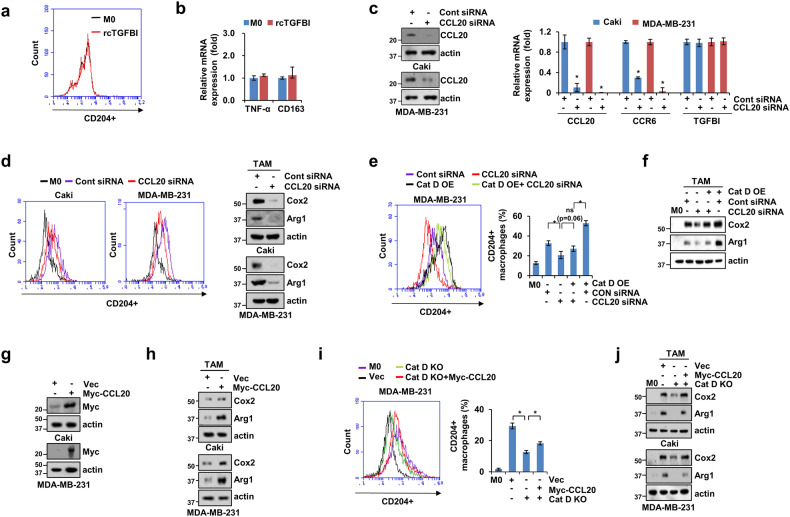
CCL20 mediates THP-1 polarization primed by Cat D-TGFBI signaling. **a**, **b** Polarization into CD204^+^ TAMs (**a**) and mRNA (**b**) expression of the THP-1 cells treated with rcTGFBI without CM. **c** CCL20 siRNA was transfected into Caki and MDA-MB-231 cells, and knockdown efficacy was verified by western blotting (left) and RT‒qPCR (right) analysis. (**d**) M0 THP-1 cells were cultured with CM and either CON siRNA- or CCL20 siRNA-transfected MDA-MB-231 and Caki cells. CD204^+^ TAMs (left) and the expression of the indicated proteins (right) were verified in polarized THP-1 cells cultured with CM. **e**, **f** MDA-MB-231 cells were cotransfected with a Cat D OE vector with either cont or CCL20 siRNA. After 48 h, the CM of the transfected MDA-MB-231 cells was collected and treated in THP-1 culture medium. CD204^+^ cells (**e**) and the expression of the indicated proteins (**f**) in THP-1 cells cultured with CM were verified. **g** The Myc-CCL20 vector was transfected into Caki and MDA-MB-231 cells, and Myc was determined by western blotting. **h** M0 THP-1 cells were cultured with CM from either Con vector- or Myc-CCL20 vector-transfected MDA-MB-231 and Caki cells. **i**, **j** MDA-MB-231 cells were cotransfected with a Cat D KO vector with either CON or Myc-CCL20 vector. After 48 h, the CM of the transfected MDA-MB-231 cells was collected and treated in THP-1 culture medium. CD204^+^ cells (**i**) and the expression of the indicated proteins (**j**) in THP-1 cells cultured with CM were determined. Error bars represent the ± SEM. *p < 0.01 in a two-sided *t* test.

To further investigate TGFBI as a negative regulator of polarization into M2-like TAMs, we treated M0 THP-1 cells with CM using rcTGFBI alone. However, rcTGFBI did not affect THP-1 polarization or the expression of TNF-α and CD163 (Fig. 7a, b). Therefore, we hypothesized that complete conditions or regulatory factors in CM from the Cat D KO cancer cells might be necessary for TAM polarization. Given the altered expression of CCL20 in the Cat D KO cancer cells, we sought to determine whether CCL20 functioned as a coregulator of Cat D-mediated TAM polarization. CCL20 was silenced using CCL20 siRNA in both Caki and MDA-MB-231 cancer cells (Fig. 7c). CM was collected from the cont siRNA- and CCL20 siRNA-transfected cancer cells and treated with M0 THP-1 cells. Lower expression of Cox2 and Arg1 and reduced CD204^+^ polarization were observed in the THP-1 cells cultured with CM from the CCL20 siRNA-transfected cancer cells compared with those from the cells cultured with CM from the cont siRNA-transfected cancer cells (Fig. 7d). To further explore the mechanisms by which Cat D suppresses M2-like TAM polarization, we transfected MDA-MB-231 cells with a Cat D overexpression vector with either CCL20 siRNA or cont siRNA, and CM from the transfected cells was used in TAM studies. Cat D overexpression strongly induced CD204^+^ polarization and Cox2 and Arg1 expression; however, combining Cat D and CCL20 siRNA nullified the effect of Cat D on CD204^+^ polarization and increased the expression of these proteins (Fig. 7e, f). In contrast, CCL20 overexpression in cancer cells resulted in an increase in Cox2 and Arg1 expression (Fig. 7g, h). In addition, we tested whether the gain-of-function of CCL20 in the Cat D KO cancer cells, CD204^+^ TAM polarization, and Cox2 and Arg1 expression were rescued in TAMs primed by CCL20 overexpression in the Cat D KO cancer cells compared with those of TAMs primed by the Cat D KO cancer cells (Fig. 7i, j). These observations suggested that Cat D regulates TAM polarization by controlling CCL20 secretion. These findings suggest that CCL20 is involved in regulating Cat D-mediated TAM polarization.

### Double deletion of Cat D and TGFBI reverts the effects of Cat D deletion in tumors

**Fig. 8:**
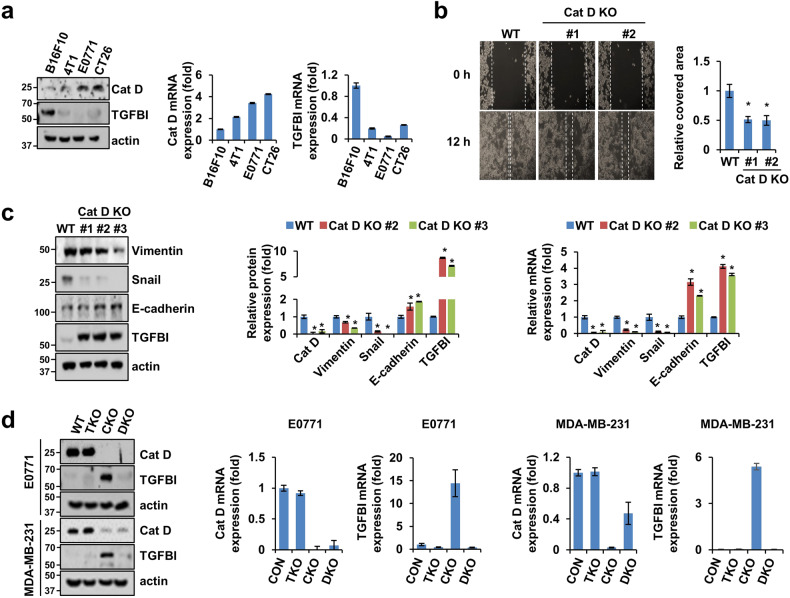
Reverse correlation between Cat D and TGFBI in mouse-derived cancer. **a** Cat D and TGFBI expression in B16F10, 4T1, E0771, and CT26 cancer cells. **b**–**c** E0771 cells were transfected with a mouse Cat D KO vector and selected using puromycin to generate a stable cell line. Migration in Cat D KO E0771 cells (**b**). Protein and mRNA expression of the indicated genes in the Cat D KO E0771 cells (**c**). **d** E0771 and MDA-MB-231 cells were transfected with either TGFBI KO or Cat D KO vector or both according to sequences of different species, mouse and human, respectively. Protein and mRNA expression of Cat D and TGFBI were verified by western blotting and RT‒qPCR, respectively. Error bars represent the ± SEM. *p < 0.01 in a two-sided *t* test.

We varied Cat D expression in various murine cancer cell lines, and an inverse correlation was observed between Cat D and TGFBI expression in B16F10, 4T1, E0771, and CT26 cancer cells (Fig. 8a). Therefore, E0771 murine breast cancer cells were used for subsequent experiments because they expressed high Cat D and low TGFBI levels. Consistent with the effects of Cat D in human cancer cells, the loss of Cat D mitigated migration in E0771 cancer cells (Fig. 8b). In addition, Cat D loss resulted in decreased vimentin and snail expression and increased E-cadherin expression in E0771 cells (Fig. 8c). To evaluate the effect of TGFBI in Cat D KO cancer cells, we generated Cat D KO (CKO), TGFBI KO (TKO), and double KO of these genes (DKO) in E0771 and MDA-MB-231 cancer cells and confirmed the protein and mRNA expression of these proteins (Fig. 8d).

**Fig. 9:**
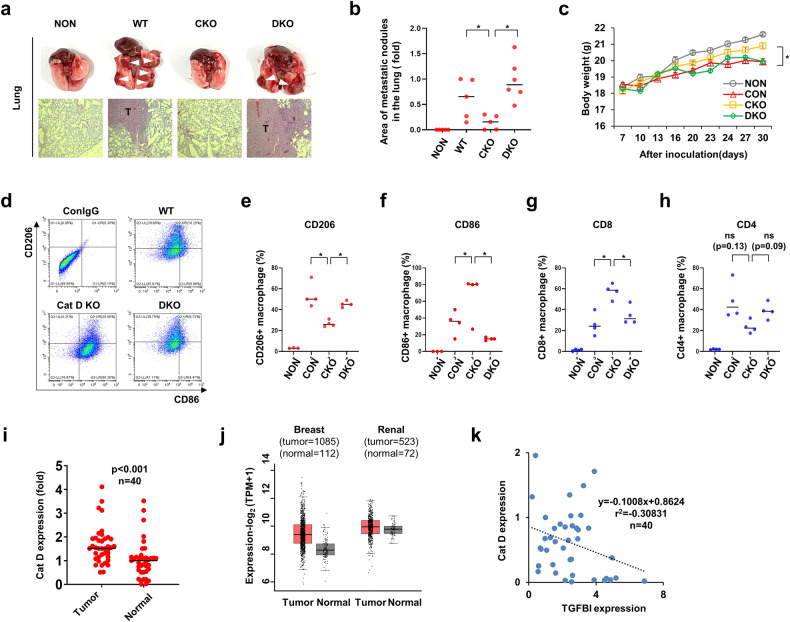
Loss of Cat D controls the population and activation of immune cells infiltrating tumors. **a**–**c** Genetically modified E0771 was inoculated into C57BL/6 mice. On Day 30 post-tail vein injection, the lung was dissected and stained with H&E (**a**). The area of the metastatic region in the lung was measured using ImageJ software (**b**). Body weight was recorded every 3–4 days (**c**). **d**–**h** On Day 20 post-subcutaneous injection, the tumor was collected, and macrophages and T cells were isolated. Flow cytometry dot plots representing tumor-infiltrating CD45^+^CD11b^+^F4/80^+^CD206^+^CD86^+^ cells (**d**). M1 (CD45^+^CD11b^+^F4/80^+^CD206^−^CD86^+^), M2 (CD45^+^CD11b^+^F4/80^+^CD206^+^CD86^−^), T cells (CD45^+^CD3^+^CD8^+^), or naive T cells (CD45^+^CD3^+^CD4^+^) were determined using FACS (**e**–**h**). **i** Cat D protein expression in normal and renal tumor tissues. **j** Cat D mRNA transcripts were analyzed using the TCGA database. **k** Correlation analysis of Cat D and TGFBI in renal cancer tissues. Error bars represent the ± SEM. *p < 0.01 by two-sided t test.

We further compared the metastatic potential of WT, CKO, and DKO E0771 breast cancer cells using the C57BL6 strain. As expected, lung metastasis of the CKO mice was mitigated compared with that of the WT mice (Fig. 9a, b). Importantly, DKO facilitated the recovery of lung metastasis, which was suppressed by CKO treatment (Fig. 9a, b). These mice also showed a significant reduction in body weight compared with the mice with CKO (Fig. 9c). This weight loss phenotype was negatively correlated with tumor metastasis in all experimental groups. Since Cat D loss is involved in suppressing M2-like TAM polarization, we speculated that it might regulate metastasis and contribute to the immune cell population and tumor activation. To ascertain this, we analyzed immune regulatory cells, such as M2, M1, and cytotoxic T-cell-infiltrated tumors, according to the exclusion gating strategy (Supplementary Fig. 5a, b). CKO diminished the population of CD45^+^CD11c^+^F4/80^+^CD206^+^CD86^−^ M2 macrophages, while it increased CD45^+^CD11c^+^F4/80^+^CD206^−^CD86^+^ M1 macrophages and CD45^+^CD3^+^CD4^−^CD8^+^ cytotoxic T cells infiltrating the tumor (Fig. 9d–h). The DKO mice exhibited reversed populations of CD206^+^CD86^−^ M2, CD206^−^CD86^+^ M1, CD4^−^, and CD8^+^ cytotoxic T cells compared with those of the CKO mice. CD4^+^ T cells did not significantly differ between the groups.

Given the importance of Cat D in regulating cancer metastasis through TGFBI-mediated TAM polarization, we assessed the clinical importance of Cat D and TGFBI and their possible relationship in human tumor tissues. Data on Cat D expression were obtained from 40 patients with renal tumors, and significantly higher Cat D expression was observed in 28 cases of tumors relative to that in normal renal tissues (Fig. 9i and Supplementary Fig. 6a). TCGA data revealed higher Cat D mRNA expression in breast and renal tumors than in their corresponding normal tissues, similar to our observation of protein expression (Fig. 9j). Furthermore, we found a negative correlation between Cat D and TGFBI expression in these tumor tissues (Fig. 9k and Supplementary Fig. 6b). Altogether, our data support the idea that Cat D deletion controls TAM polarization from immune-suppressive M2 to immune-stimulatory M1 subtypes through TGFBI and CCL20 secretion, which in turn leads to suppression of tumor metastasis and growth.

## Discussion

**Fig. 10:**
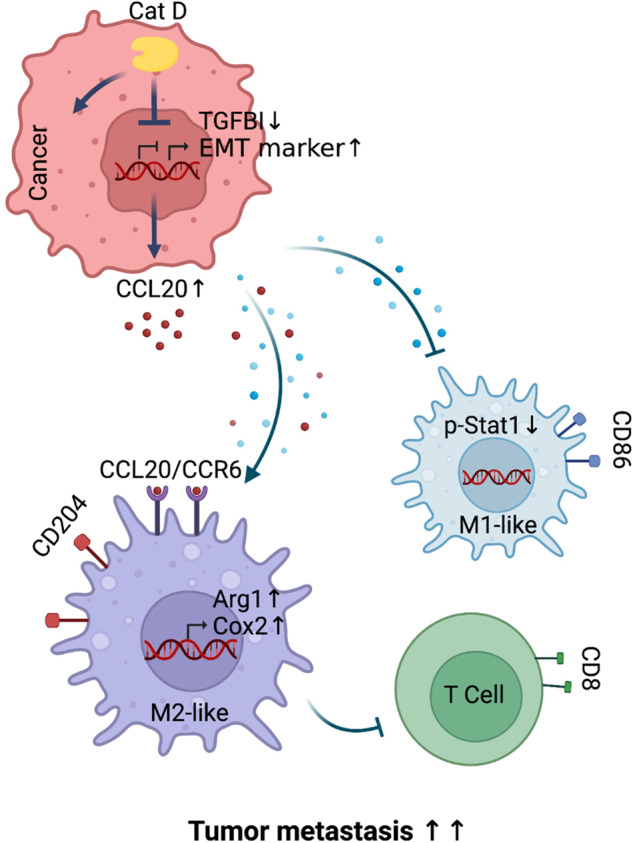
Scheme showing an important role of Cat D in the metastatic potential of tumors through TGFBI-CCL20-mediated TAM polarization. Loss of Cat D suppressed polarization into M2-like TAMs through upregulation of TGFBI and downregulation of CCL20. In turn, macrophages primed using Cat D KO cancer cells remarkably suppressed cancer metastasis. Altogether, controlling the activity and expression of Cat D can be considered a potential combination therapy for treating cancer metastasis and immune escape. Created with BioRender.com.

Although the role of Cat D in various types of cancers has been extensively investigated^22,23^, the immunomodulatory function and the exact molecular mechanisms of this molecule underlying effects in the TME are completely unknown. In this study, we revealed that Cat D is a critical regulator of EMT and the metastatic potential of tumors. Our loss-of-function data proved that Cat D is a positive regulator of EMT in cancer and showed an inverse correlation with TGFBI expression in various types of cancer cells. In fact, even though Cat D KO cancer cells resulted in a reduction in metastatic potential in NOD-SCID mice, an immunodeficient mouse strain, TGFBI blockade failed to or marginally recovered the reduced metastatic potential of the Cat D KO cancer cells. These data suggest that TGFBI may not provide sufficient signals to Cat D KO cancer cell metastasis in immunodeficient mice. In contrast, in the C57BL/6 strain, Cat D and TGFBI double deletion completely reversed the anti-metastatic effect of the Cat D KO cancer cells. Thus, we investigated whether TGFBI could affect metastatic potential by regulating other cell types present in Cat D KO tumor tissues. Herein, we proved that, in addition to controlling cancer cells, the loss of Cat D-mediated TGFBI expression was closely involved in TME control (Fig. 10).

Previous studies have reported that TGFBI is secreted from fibroblasts and cancer, endothelial, and immune cells and regulates their activity and function^24–26^. Moreover, TAM-derived TGFBI promotes glioblastoma growth through integrin αvβ5-Src-Stat3 signaling^27^. TGFBI also functions in cancer cells in an autocrine manner; however, its effects remain controversial and seem context-dependent^28^. Some studies have reported a positive correlation between TGFBI expression and the polarization of macrophages toward the M2 subtype, which in turn promotes tumor progression and metastasis^24,25^. The upregulation of TGFBI induced by Cat D is negatively correlated with M2 polarization and the metastatic potential of tumors. Cat D KO cancer cells inhibited polarization into CD204^+^ M2-like TAMs. Furthermore, CD204^+^ TAMs primed using parent cancer cells were reduced following TGFBI protein treatment. In contrast, TGFBI blockade recovered the CD204^+^ TAM polarization induced by Cat D KO cancer cells. Furthermore, Cox2 and Arg1 expression in these cells was positively correlated with the M2-like TAM population, consistent with previous reports^29,30^. These results suggest that Cat D contributes to both EMT and TAM polarization. In particular, Cat D-mediated TAM polarization is regulated by TGFBI. Given this finding, we postulated that TGFBI plays a biphasic role within the TME, and its functions are heavily dependent on the cellular and tumor context. Furthermore, our investigations have revealed that both Cat D mutants and deletions have regulatory effects on mRNA expression, in addition to protein expression. These findings suggest that a specific transcription factor may be involved in the regulation of TGFBI expression under conditions of Cat D KO. Consequently, further studies are warranted to elucidate the identity of this transcription factor and its role in either repressing or promoting TGFBI expression in renal and breast cancer.

Cat D inhibition leads to anti-inflammatory effects in bone marrow-derived macrophages by inhibiting TNF-α and CCL2 expression under LPS-stimulated conditions^31^. Cancer cells are capable of producing several cytokines and predominantly control the differentiation and activation of immune cells, including TAM polarization^32,33^. The CCL20/CCR6 axis enhances tumor growth and metastasis, and its overexpression correlates with CD163^+^ macrophages in tongue squamous cell carcinoma^34^. We observed decreased CCL20 expression in Cat D KO cancer cells, which increased following TGFBI blockade. Functionally, CCL20 knockdown in cancer suppressed M2-like TAM polarization when cultured with CM, whereas CCL20 overexpression suppressed polarization. Cat D-mediated TAM polarization is critically regulated by CCL20 expression, as verified by gain- and loss-of-function studies. The lack of an effect of TGFBI treatment on TAM polarization under culture conditions without CM further highlighted the biological necessity of TGFBI-mediated CCL20 in CM from Cat D KO cancer cells. These data suggest that CCL20 is a major downstream target of Cat D-TGFBI signaling in tumors, eventually supporting TAM polarization. Thus, we suggest that M2-like TAM polarization is suppressed due to cytokine/chemokine secretion in the cancer microenvironment of Cat D KO cancer cells.

Natural killer (NK) and CD8^+^ T cells are strong cytotoxic immune cells involved in the anti-cancer immune response^35^. The sensitivity and cytokine production of these immune cells are regulated by TAMs, and thus, interactions between TAMs and cytotoxic immune cells are considered a key determinant in tumor progression and metastasis^36^. Several previous studies have demonstrated that lysosomal proteases play an important role in the TME^37,38^. Cathepsins B, L, and S reportedly play key roles in macrophage function, particularly in M2 macrophage function^38^. Therefore, we deduced that immune cell-derived Cat D may also be associated with their functional activities as well as cancer, although we have no details regarding the effects of Cat D on other immune cell types. In the present study, we observed that TAMs were skewed toward the M1 phenotype and increased the cytotoxicity of T cells in Cat D KO tumors. Importantly, Cat D and TGFBI double KO tumors tended to exhibit a decrease in cytotoxic T cells and an increase in M2-like TAMs compared with those of Cat D KO tumors. Thus, we suggest that Cat D-TGFBI is necessary for regulating immune cell activation to promote the anti-tumorigenic and anti-metastatic immune status, indicating increased sensitivity to chemo- and immunotherapy. However, notably, sensitivity in response to chemo- and immunotherapy of Cat D KO tumors was not assessed in the present study. Therefore, details of the regulation of drug sensitivity by TAM polarization mediated by Cat D KO need to be further addressed. Furthermore, we found higher Cat D expression in human tumor tissues than in adjacent normal tissues. In fact, 12 of 40 cases (30%) showed no difference or lower expression of Cat D in tumor tissues than in normal tissues. More importantly, a significant negative correlation between Cat D and TGFBI was observed in most tumor cases, suggesting that Cat D and TGFBI have crucial clinical value and may serve as cancer biomarkers.

We revealed an important role of Cat D in the metastatic potential of tumors through TGFBI-CCL20-mediated TAM polarization. Loss of Cat D suppressed polarization into M2-like TAMs through upregulation of TGFBI and downregulation of CCL20. In turn, macrophages primed using Cat D KO cancer cells substantially suppressed cancer metastasis. Altogether, controlling the activity and expression of Cat D can be considered a potential combination therapy for treating cancer metastasis and immune escape.

### Supplementary information

Supplementary Information

Supplementary Table
