# Supplementary material for: Cathepsin D promotes polarization of tumor-associated macrophages and metastasis through TGFBI-CCL20 signaling
Source: Exp Mol Med. 2024 Feb 1;56(2):383–94. doi: 10.1038/s12276-024-01163-9 (PMC10907383; doi:10.1038/s12276-024-01163-9)
Supplement: Supplementary file 1 — Supplementary Information [file 12276_2024_1163_MOESM1_ESM.pdf]

Supplementary figures and table

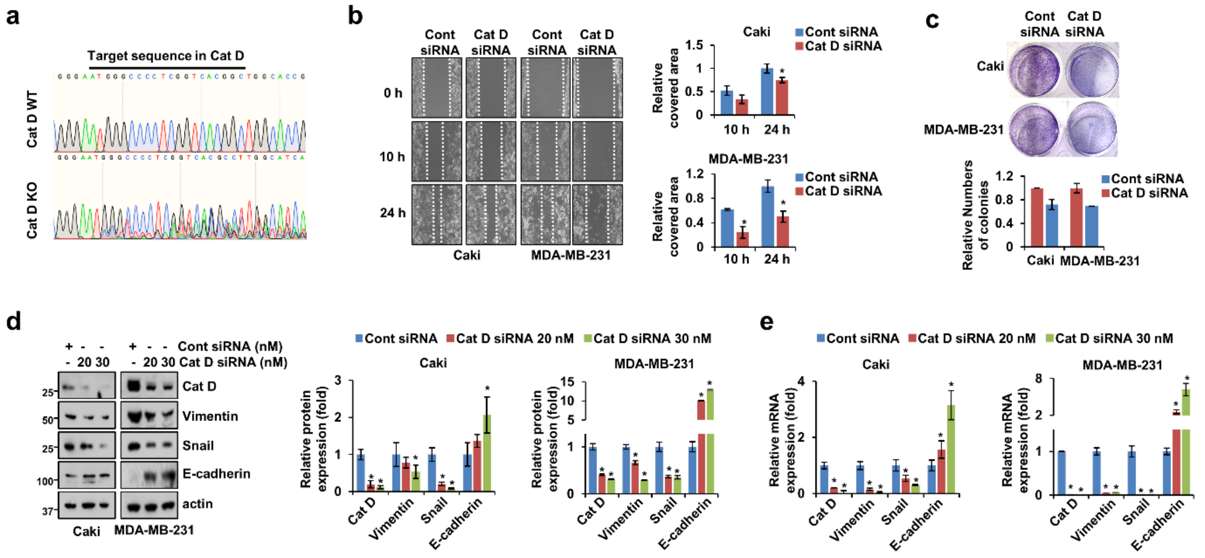

Supplementary Fig. 1 Knockdown of Cat D inhibits EMT in Caki and MDA-MB-231 cancer cells. **a** Cat D KO in Caki cancer cells is verified by sequencing. **b,c** Migration (b) and colony formation (c) assays were performed for CON and Cat D siRNA transfected Caki and MDA-MB-231 cells. **d-e** The protein (d) and mRNA (e) expression of the indicated genes in Caki and MDA-MB-231 transfected cells. Analysis of quantification was performed using ImageJ software. Error bars represent the ± SEM. $*p < 0.01$ in a two-sided *t*-test

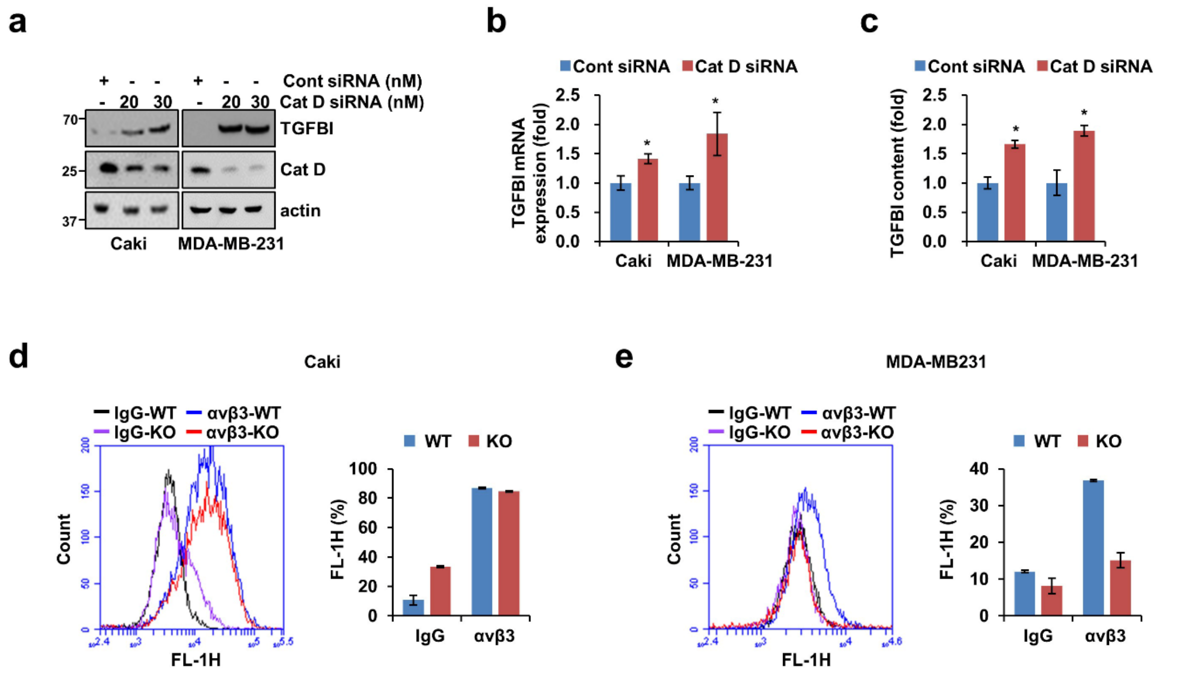

Supplementary Fig. 2 Knockdown of Cat D positively regulates TGFBI expression. a-c Representative western blot (a) images of CON and Cat D siRNA-transfected Caki and MDA-MB-231 cancer cells. TGFBI mRNA (b) and secretory levels (c) were verified by RT-qPCR and ELISA, respectively. **d,e** avb3 expression in WT and Cat D KO Caki (d) and MDA-MB-231 (e) cancer cells were detected using FACS. Error bars represent the ± SEM. *$p < 0.01$ in a two-sided t-test.

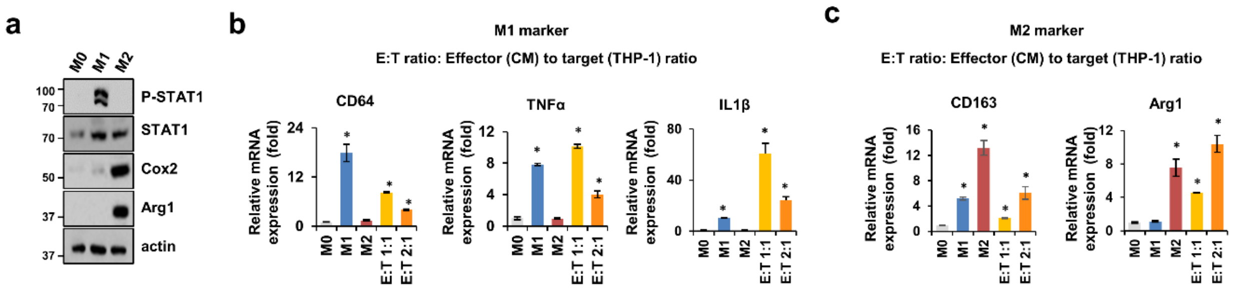

Supplementary Fig. 3 Expression of M1- and M2-macrophage-related genes under CM culture conditions. a-c. THP-1 cells were treated with LPS/IFNγ (referred to in the text as "M1") or IL-4/IL-13 (referred to in the text as "M2"). CM was prepared in Caki culture medium concentrated for 2 days, and the CM was used as an effector (as indicated "E") and then treated in unpolarized M0 THP-1 (as indicated "T) culture medium. The protein expression of the indicated genes in the differentiated cells (a). mRNA expression of the indicated genes in CM-treated THP-1 cells at different dilution rates (b, c). Error bars represent the ± SEM. *$p < 0.01$ compared with M0.

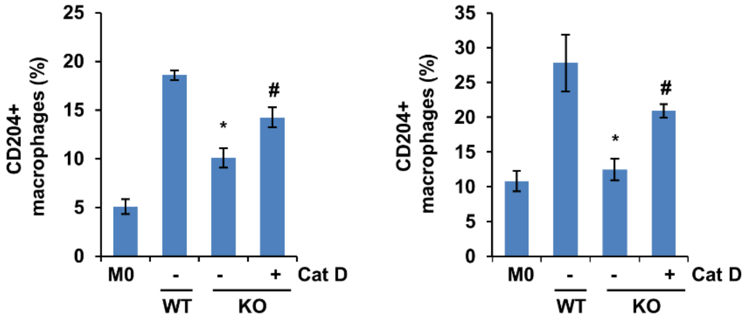

Supplementary Fig. 4 Restored Cat D induces THP-1 polarization into an M2-like phenotype. Control and Cat D OE vectors were transfected into Caki and MDA-MB-231 cancer cells and cultured for 2 days. CM was collected from these cancer cells to induce THP-1 polarization, as shown by FACS analysis. Error bars represent the ± SEM. $^{*}p < 0.01$ compared with control cells. $^{\#}p < 0.01$ compared with KO cells.

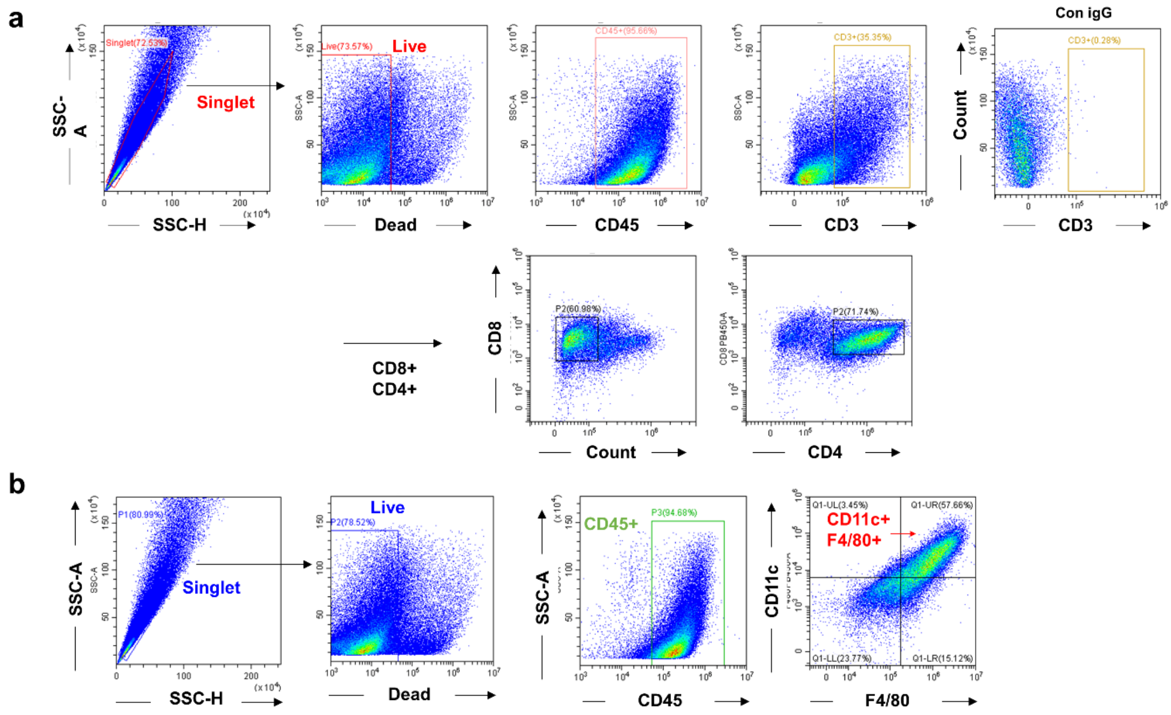

Supplementary Fig. 5 Isolation of macrophages and T cells in primary tumor. a, b. Gating strategy for T-cell isolation in tumors. Primary macrophages and T cells in an E0771 tumor were sorted using FACS. T cells were gated with CD45/CD3 fluorescence, and cytotoxic $CD8^+$ or naïve $CD4^+$ T cells were determined. Macrophages were gated with CD45/CD11c/F4/80 fluorescence and $CD206^+$ or $CD86^+$ macrophages were determined.

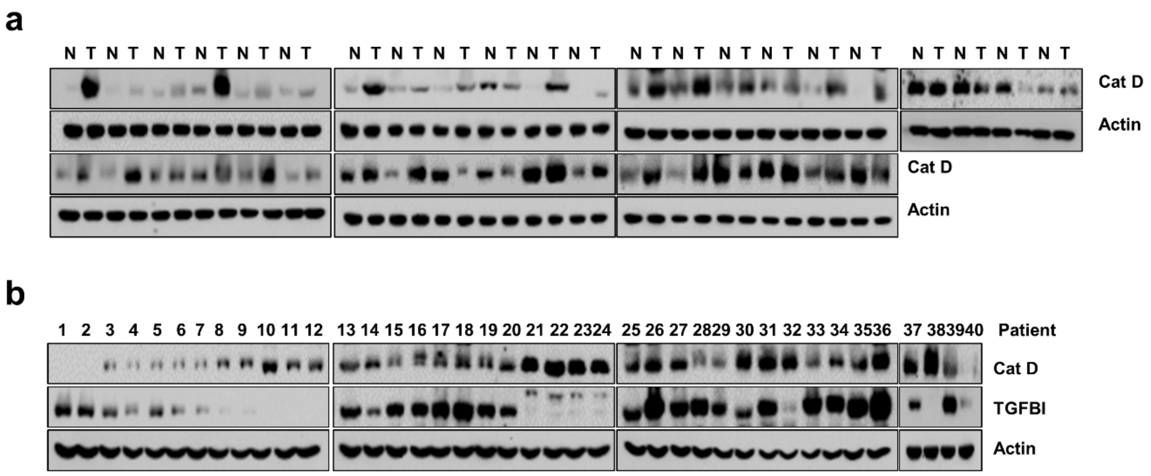

Supplementary Fig. 6 Cat D and TGFBI in renal tumor tissues are negatively correlated.

a The expression of Cat D in paired renal tumor tissues and the corresponding normal tissues (*n* = 40). **b** The expression of Cat D and TGFBI in renal tumor tissues.
